# Sophorolipid protects against early-weaning syndrome by improving the gut microenvironment in early-weaned piglets

**DOI:** 10.1186/s12917-021-03105-3

**Published:** 2022-01-03

**Authors:** Min-Jin Kwak, Sun-Woo Choi, Yong-Soon Choi, Hanbae Lee, Kwang-Youn Whang

**Affiliations:** 1 Department of Biotechnology, Korea University, 145 Anam-ro, Seoul, 02841 Republic of Korea; 2 Division of Interdisciplinary Program in Precision Public Health (BK21 FOUR Program), Department of Biomedical Engineering, Korea University, Seoul, 02841 Republic of Korea; 3 Pathway Intermediates, Seoul, 02841 Republic of Korea

**Keywords:** Pig, Gut microbiota, Gut morphology, Local inflammation, Mucus, Sophorolipids

## Abstract

**Background:**

In animals, weaning stress is the first and most critical stress. Weaning can negatively affect the growth performance of animals physically, psychologically, and pathologically. Our previous studies on the HT-29 cell line and early-weaned rats demonstrated that adequate sophorolipid (SPL) supplementation in feed could enhance the mucin-producing and wound healing capacities of the gut defense system by modulating gut microbiota.

**Methods:**

We conducted an experiment with one hundred forty 21-day-old early weaned piglets (L x Y x D). They were allocated into 4 treatment and 7 replications (4 pigs per pen) according to their initial body weight. Body weight and feed intake were measured biweekly during experimental period. After 6 weeks, 28 pigs were randomly selected and sacrificed to collect plasma, jejunum, and cecal content samples.

**Results:**

Dietary SPL supplementation at 5 and 10 mg/kg quadratically increased the average daily gain during the experimental period in the treatment groups when compared with the control group. The albumin levels of piglets fed with the SPL supplemented diet were downregulated to the normal range. Moreover, in feed, SPL supplementation at 5 and 10 mg/kg improved jejunal histological indices and gene expression levels related to mucin secretion and local inflammation markers. Consistent with these results, adequate SPL supplementation (5 and 10 mg/kg) increased the population of *Prevotella*, a beneficial bacterium, and its short-chain fatty acid production in the ceca of piglets.

**Conclusions:**

The occurrence of diarrhea after weaning in piglets could be reduced by feeding a 10 ppm of SPL supplemented diet which improves the gut defense system by improving the microbial population and enhancing mucin layer integrity.

## Background

Weaning is the transitional phase of feeding in mammals from reliance on the mother’s milk to an adult diet. This process is stressful for mammals because changing feed form could result in wounds in the digestive tract, especially in the villi [1]. Neuenschwander et al. [2] demonstrated that weaning significantly decreased growth performance and nutrient digestibility. To solve these problems, the use of sub-therapeutic dosages of antibiotic growth promoters has been widely adopted in the livestock industry owing to their outstanding efficacy in feed conversion and animal growth [3], however, antibiotic growth promoters have been banned because of the increasing risk of the microbial resistome [4]. Therefore, the livestock feed industry seeks alternatives to antibiotics, including organic and inorganic acids, enzymes, pro- and prebiotics, phytochemicals, and nano-compounds [5, 6]. Nevertheless, various alternatives to antibiotic growth promoters have not been able to keep up with the growth-accelerating effect of sub-therapeutic dosage of antibiotics [7].

In recent, bio-emulsifier molecules have been attracted attention due to their antimicrobial properties similar to antibiotic growth promoters [8]. Among bio-emulsifiers, sophorolipid (SPL) has emerged as a substitute for antibiotics owing to its relatively low toxicity and high biodegradability [9, 10], and it have been already adjusted in various industry, including medicine, hygiene, and pharmaco-dermatology [11]. SPL is a glycolipid emulsifier, produced by non-pathogenic yeast, and it contains a dimeric sugar and hydroxyl fatty acid linked by a glycosidic bond [12]. SPL displays various unique biological properties, including immunomodulation, skin dermal fibroblast stimulation, and collagen production [13]. Moreover, SPLs exhibit antimicrobial, anticancer, antiviral, and antifungal properties as well as cytotoxicity [14, 15]. These properties imply that SPLs can be applied in fields such as medicine, hygiene, and pharmaco-dermatology. However, despite their considerable potential, the use of SPLs in the animal feed industry has not yet been reported.

Therefore, the present study aimed to determine the effects of dietary SPL supplementation on the gastrointestinal health in weaned piglets. To this effect, we conducted a dietary experiment with early-weaned piglets a concentration of 5, 10, and 15 mg/kg according to previous dosage-determining experiments in piglets.

## Methods

### Piglets and diets

All of works related to animal was conducted in accordance with the guidelines and regulations for the care and the use of experimental animals was approved by the Korea University Institutional Animal Care & Use Committee (Permission No. KUIACUC-2021-0021). We conducted all animal studies in accordance with the guidelines and regulations of the Animal Ethics Committee approved by Korea University (Seoul, Republic of Korea). The experiment was performed at a clean, controlled research farm in Cheonan, Republic of Korea, and pigs were supplied from the commercial farm in Sejong, Republic of Korea. A total of 140 early-weaned piglets (L × Y × D; 21 days old) were randomly allotted to four treatments according to their body weight (BW; initial mean BW: 6.57 kg). A pen was an experimental unit and each treatment had seven replications (5 pigs per pen). The dietary treatments consisted of CON (basal diet), SPL5 (5 mg/kg SPL supplementation), SPL10 (10 mg/kg SPL supplementation), and SPL15 (15 mg/kg SPL supplementation). The feed composition and calculated nutritional values are shown in Table 1. The feed and SPL were supplied by EASY BIO Inc. (Seoul, Republic of Korea). Feed and water were supplied ad libitum to the pigs during the experiment. The raising program consisted of three phases: phase 1, day 1–7; phase 2, day 8–21; phase 3, day 22–42. The BW and feed intake were measured biweekly to calculate average daily gain (ADG), average daily feed intake (ADFI), and feed efficiency (FE).

**Table 1:** Composition and nutritional value of basal diets^a^

Ingredients, %	Phase 1	Phase 2	Phase 3
Corn	47.80	53.90	62.40
Soybean meal	5.00	20.15	24.20
Fish meal	4.50	4.00	3.00
Plasma powder	5.00	2.50	0.00
Whey powder	20.00	10.00	5.00
Lactose	8.00	3.00	0.00
Soybean oil	5.50	2.50	2.00
Lysine sulfate	0.38	0.46	0.32
Methionine	0.31	0.24	0.16
Threonine	0.15	0.15	0.10
Tryptophane	0.05	0.05	0.02
Choline chloride	0.10	0.05	0.05
MCP	0.86	1.17	0.60
Limestone	1.52	1.00	1.33
Salt	0.20	0.20	0.20
Zinc oxide	0.28	0.28	0.28
Vitamin premix^b^	0.12	0.12	0.12
Mineral premix^c^	0.15	0.15	0.15
Phytase	0.03	0.03	0.03
NSPase	0.05	0.05	0.05
Total	100.00	100.00	100.00
Calculated value			
ME (kcal/ kg)	3673.00	3544.00	3467.00
CP (%)	20.63	19.80	18.80
Calcium (%)	0.98	0.75	0.75
Phosphate (%)	0.65	0.68	0.50
Lysine (%)	1.60	1.41	1.22
Methionine (%)	0.62	0.55	0.46
Threonine (%)	1.07	0.95	0.83
Tryptophan (%)	0.32	0.29	0.24

^a^*Abbreviations*: *CP* crude protein, *MCP* monocalcium phosphate, *ME* metabolizable energy, *NSPase* non-starch polysaccharidase

^b^Provided per kilogram of complete diet: vitamin A, 12,000 IU; vitamin D_3_, 2500 IU; vitamin E, 30 IU; vitamin K_3_, 3 mg; pantothenic acid, 15 mg; nicotinic acid, 40 mg; choline, 400 mg; vitamin B_12_, 12 μg

^c^Provided per kilogram of complete diet: iron, 90 mg; copper, 8.8 mg; zinc, 100 mg; manganese, 54 mg; iodine, 0.35 mg; selenium, 0.30 mg

### Sample collection

At the end of the experiment, 28 piglets (one piglet per pen, randomly selected) were sacrificed with a captive bolt and exsanguinated. Blood samples were collected from the jugular vein into heparin-coated plasma tubes (BD Vacutainer; Beckton Dickinson Rowa Denmark, Kongens Lyngby, Denmark) for analysis of biochemical markers (glucose, triglyceride, total cholesterol, blood urea nitrogen, albumin, and creatinine). Thereafter, jejunal and cecal samples were obtained, immediately frozen using dry ice, and stored at − 80 °C until further analysis. In addition, 10 mm of central parts of the jejunum were fixed in a 4% formalin solution for histological analysis. The farm owner allowed all tissue samples to be collected.

### Blood biochemical markers

The concentrations of glucose, triglyceride, total cholesterol, blood urea nitrogen, albumin, and creatinine in the plasma samples were determined using commercial kits (EMBIELTM, Seoul, Korea) according to the manufacturer’s instructions. The absorbance of samples was measured using a spectrophotometer (Zenyth 200rt; Biochrom, Berlin, Germany) at a specific wavelength, and the concentration of samples was calculated using the standard curve of each biomarker.

### Histological analysis of the jejunum

The fixed jejunum samples were embedded into paraffin blocks, and 5 μm cross-sections were prepared using a rotary microtome (CUT 5062; SLEE Medical, Mainz, Germany). The jejunum sections were then stained with hematoxylin and eosin and Alcian blue. Subsequently, a total of 15 villi and 15 crypts were randomly selected per experimental unit, and a single observer measured the villus height and crypt depth, and counted the number of goblet cells.

### RNA extraction from the jejunum and cecum

Trizol® (Invitrogen, Grand Island, NY, USA) was used to extract total RNA from jejunal and cecal samples, and a Nanodrop spectrophotometer (Thermo Fisher Scientific, Wilmington, DE, USA) was used to determine the concentration and purity of extracted RNA. Consequently, a High-Capacity cDNA Reverse Transcription kit (Applied Biosystems, Carlsbad, CA, USA) was used to synthesize cDNA samples according to the manufacturer’s instructions.

### RNA analysis and cecal bacteria analysis by qRT-PCR

Gene expression levels of inflammatory cytokines (interleukin-8, IL-8; interferon-γ, IFN-γ; tumor necrosis factor-α, TNF-α; and interleukin-10, IL-10) and tight junction proteins (zonula occludens-1, ZO-1; occludin, OCLD; and claudin 1, CLDN1) in jejunal samples were determined by qRT-PCR using a RealHelixTM Premier qPCR kit (NanoHelix, Daejeon, Korea) with a StepOnePlus Real-Time PCR System (Applied Biosystems). Additionally, the expression levels of mucin 2 (MUC2) were also determined in both the jejunum and cecum. Glyceraldehyde-3-phosphate dehydrogenase (GAPDH) was used as a housekeeping gene. The 2^-ΔΔCT^ method was used to quantify relative mRNA expression levels. The primers for the target genes are listed in Table 2.

**Table 2:** Oligonucleotide primers used in qRT-PCR analysis^a^

Gene name	Sequence (forward, reverse)	Reference
Housekeeping gene		
GAPDH	F: 5′- GAGGTCGGAGTGAACGGAT −3’	[16]
	R: 5′- CCTGGGTCGAATCATACTGGAACA − 3′	
Inflammatory cytokines		
IL-8	F: 5′- TTTCTGCAGCTCTCTGTGAGG −3’	[17]
	R: 5′- CTGCTGTTGTTGTTGCTTCTC − 3′	
IFN-γ	F: 5′- GTTTTTCTGGCTCTTACTGC − 3’	[18]
	R: 5′- CTTCCGCTTTCTTAGGTTAG − 3′	
TNF-α	F: 5′- ATCGGCCCCCAGAAGGAAGAG − 3’	[19]
	R: 5′- GATGGCAGAGAGGAGGTTGAC − 3′	
IL-10	F: 5′- GCATCCACTTCCCAACCA − 3’	[20]
	R: 5′- CTTCCTCATCTTCATCGTCAT − 3′	
Tight junction proteins		
ZO-1	F: 5′- AAGCCCTAAGTTCAATCACAATCT − 3’	[21]
	R: 5′- ATCAAACTCAGGAGGCGGC − 3′	
OCLD	F: 5′- TCCTGGGTGTGATGGTGTTC − 3’	[21]
	R: 5′- CGTAGAGTCCAGTCACCGCA − 3′	
CLDN1	F: 5′- TCGACTCCTTGCTGAATCTG − 3’	[16]
	R: 5′- TTACCATACCTTGCTGTGGC − 3′	
Mucin production		
MUC2	F: 5′- GGCTGCTCATTGAGAGGAGT − 3’	[22]
	R: 5′- ATGTTCCCGAACTCCAAGG − 3’	

^*a*^*Abbreviations*: *CLDN1* claudin-1, *GAPDH* glyceraldehyde-3-phosphate dehydrogenase, *IFN-γ* interferon-γ, *IL-8* interleukin-8, *IL-10* interleukin-10, *MUC2* mucin 2, *OCLD* occludin, *TNF-α* tumor necrosis factor- α, *ZO-1* zona occludens 1

### Cecal gDNA extraction and quantification

DNA was extracted using a DNeasyPowerSoil Kit (Qiagen, Hilden, Germany) according to the manufacturer’s instructions. The extracted DNA was subsequently quantified using Quant-IT PicoGreen (Invitrogen).

### Library construction and sequencing

The sequencing libraries were prepared according to the Illumina 16S Metagenomic Sequencing Library protocols to amplify the V3 and V4 regions. The input gDNA, 2 ng, was PCR amplified using 5× reaction buffer, 1 mM dNTP mix, 500 nM each of the universal F/R PCR primers, and Herculase II fusion DNA polymerase (Agilent Technologies, Santa Clara, CA, USA). The cycling conditions for the first PCR were 3 min at 95 °C for heat activation, and 25 cycles of 30 s at 95 °C, 30 s at 55 °C, and 30 s at 72 °C, followed by a 5 min final extension at 72 °C. The universal primer pair with Illumina adapter overhang sequences used for the first amplification were as follows: V3-F: 5′-TCGTCGGCAGCGTCAGATGTGTATAAGAGACAGCCTACGGGNGG CWGCAG-3′, V4-R: 5′- GTCTCGTGGGCTCGGAGATGTGTATAAGAGACAGGACTAC HVGGGTATCTAATCC-3′. The first PCR product was purified using AMPure beads (Agencourt Bioscience, Beverly, MA, USA). Following purification, 2 μL of the first PCR product was PCR amplified for final library construction containing the index using NexteraXT Indexed Primer. The cycling conditions of the second PCR were the same as those for the first PCR, except those 10 cycles were run. Thereafter, the PCR product was purified using AMPure beads. The final purified product was then quantified using qPCR according to the qPCR Quantification Protocol Guide (KAPA Library Quantification kits for Illumina Sequencing platforms) and qualified using the TapeStation D1000 ScreenTape (Agilent Technologies Deutschland GmbH, Waldbronn, Germany). Paired-end (2 × 300 bp) sequencing was performed at Macrogen using the MiSeq™ platform (Illumina, San Diego, CA, USA).

### Cecal short-chain fatty acids (SCFA) measurement

Gas chromatography-mass spectrometry (GC-MS) is used to determine the concentration of short-chain fatty acids (SCFA) in the cecal contents according to the method suggested by Furusawa [23]. SCFA concentrations were quantified by comparing their peak areas with the standards.

### Statistical analysis

Growth performance was analyzed using the GLM procedures of SAS (1989), and polynomial contrasts (linear, quadratic, and cubic) were used to test the effect of SPL supplementation levels. The other physiological parameters (serum biochemical markers, gut histological data, gene expression levels in the jejunum and cecum, gut microbial population, and cecal SCFA concentrations) were analyzed using analysis of variance. All data analyses were conducted using SAS 9.4 (SAS Institute, Cary, NC, USA). Significant differences between the treatments were determined using Duncan’s multiple range tests at a *p* < 0.05 level of significance.

## Results

### Growth performance

At the end of the experiment, the piglets in the SPL5 and SPL10 treatment groups were 1.84 and 1.16% heavier (Table 3), respectively, than those in the CON group, although the difference was not statistically significant (*p* > 0.05). Dietary 15 ppm of SPL didn’t change body weight compared to CON group. During the experimental period, dietary SPL supplementation quadratically increased the ADG of piglets fed with the experimental diets in a dose-dependent manner (*p* < 0.05). However, ADFI and FE of pigs fed the SPL-supplemented diet did not differ among the treatments.

**Table 3:** Growth performance of weaning pigs fed experimental diets

	Dietary SPL levels (ppm)					Polynomial contrast^b^		
	0	5	10	15	SEM^a^	L	Q	C
Body weight, kg								
Week 0	6.56	6.58	6.60	6.57	0.24	0.990	0.978	0.970
Week 2	10.16	10.15	10.19	10.08	0.25	0.918	0.924	0.908
Week 4	16.34	16.49	16.51	16.27	0.26	0.926	0.718	0.945
Week 6	24.97	25.43	25.26	24.82	0.28	0.825	0.465	0.901
Overall								
ADG, g	438.26	448.91	444.21	434.39	2.55	0.446	0.048	0.621
ADFI, g	598.14	602.64	589.87	591.21	4.36	0.410	0.881	0.430
FE	0.733	0.746	0.754	0.736	0.006	0.777	0.219	0.684

^a^ Standard error of mean

^b^ *L* linear, *Q* quadratic, *C* cubic

### Serum biochemical analysis

The concentrations of glucose, triglyceride, total cholesterol, blood urea nitrogen, and creatinine were not changed by three dosages of SPL supplementation in feed (Table 4). However, the albumin concentration of piglets in the CON group was significantly higher than that for piglets in the dietary SPL-supplemented groups (*p* < 0.05).

**Table 4:** Biochemical markers of weanling pigs fed experimental diets

	Dietary SPL levels (ppm)					
	0	5	10	15	SEM^a^	*p*-value
Serum biochemical markers, mg/dl						
Glucose	67.08	66.59	68.50	65.92	3.588	0.997
Triglyceride	186.14	180.38	176.63	178.93	8.755	0.988
Total cholesterol	86.47	85.27	83.95	83.11	1.470	0.886
Blood urea nitrogen	5.22	5.02	5.12	4.44	0.331	0.863
Albumin	4.22^a^	3.84^b^	3.51^b^	3.80^b^	0.078	0.005
Creatinine	0.82	0.81	0.81	0.84	0.006	0.162

^a^standard error of means

### Gut histological analysis

**Fig. 1:**
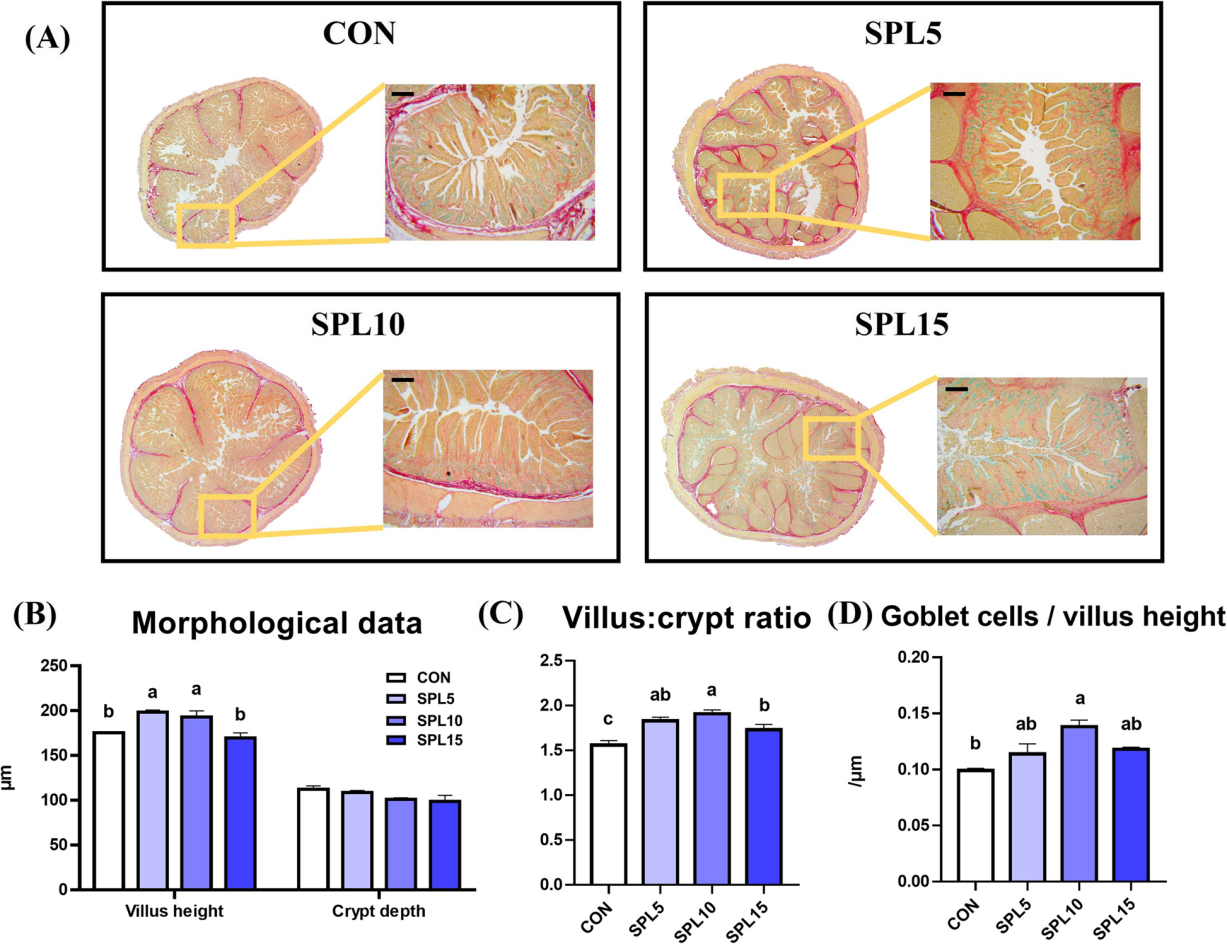
Gut morphological data from histological analysis of pigs fed experimental diets. **A** Representative pictures of jejunum of pigs; **B-C** Morphological indexes (villus height, crypt depth, and their ratio) of pigs; **D** The number of goblet cells per villus height in jejunum of pigs. ^a,b,c^ Mean values within a row have different superscript letters were significantly different (*P* < 0.05). Treatment groups: CON, control group fed with basal diet; SPL5, group fed with 5 mg/kg of SPL supplemented diet; SPL10, group fed with 10 mg/kg of sophorolipid-supplemented diet; SPL15, group fed with 15 mg/kg of sophorolipid-supplemented diet

Representative images of the jejunum stained by Alcian blue are presented in Fig. 1A. The SPL5 and SPL10 groups had significantly increased villus height compared to CON and SPL15 groups, and piglets fed all dosages of SPL supplemented diets showed a significantly higher villus: crypt ratio compared with piglets in the CON group (*p* < 0.05; Fig. 1B and C). Additionally, the number of goblet cells per villus height was significantly increased in the SPL10 treatment relative to the other treatments (*p* < 0.05; Fig. 1D).

### Gene expression levels in the gastrointestinal tract

**Fig. 2:**
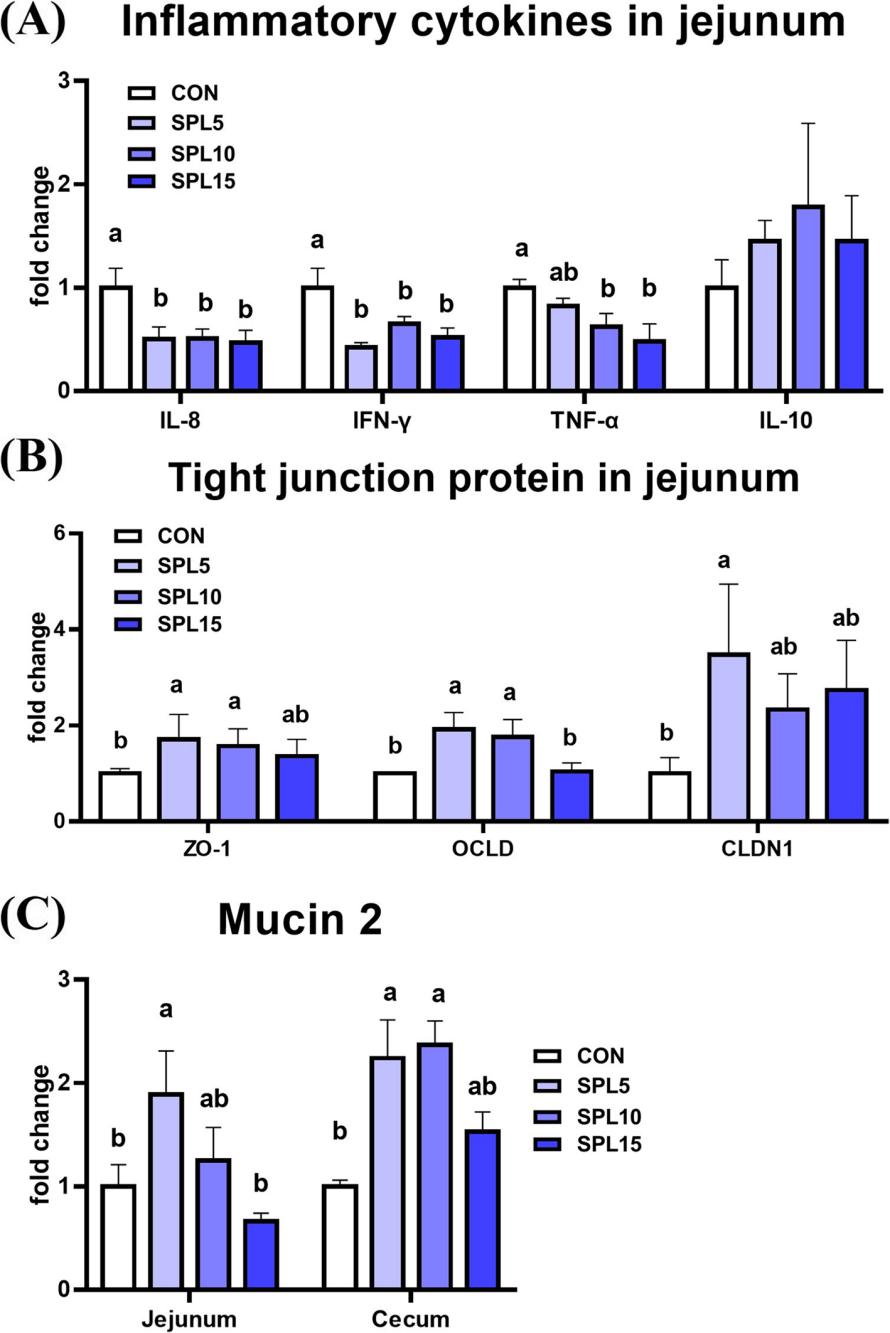
mRNA expression levels of genes in jejunum and cecum of pigs fed experimental diets. **A** Genes related to inflammation cytokines (IL-8, IFN-γ, TNF-α, and IL-10); **B** Genes related to tight junction proteins (ZO-1, OCLD, and CLDN1); C) MUC2 expression in jejunum and cecum. ^a,b^ Mean values within a row have different superscript letters were significantly different (*P* < 0.05). Treatment groups: CON, control group fed with basal diet; SPL5, group fed with 5 mg/kg of SPL supplemented diet; SPL10, group fed with 10 mg/kg of sophorolipid-supplemented diet; SPL15, group fed with 15 mg/kg of sophorolipid-supplemented diet. Abbreviations: IL-8, interleukin-8; IFN-γ, interferon-γ; TNF- α, tumor necrosis factor- α; IL-10, interleukin-10; ZO-1, zona occludensa-1; OCLD, occludin; CLDN1, claudin-1; MUC2, mucin 2

In the jejunum, all dosages of SPL supplementation significantly reduced the expression levels of pro-inflammatory cytokines IL-8, TNF-α, and IFN-γ when compared to the CON group (*p* < 0.05) without modulation of the anti-inflammatory cytokine IL-10 (Fig. 2A). Additionally, the expression of genes related to tight junction proteins (ZO-1, OCLD, and CLDN) was significantly upregulated in the SPL5 and SPL10 groups when compared to the CON and SPL15 groups (*p* < 0.05; Fig. 2B). Moreover, the expression level of MUC2 in the jejunum and cecum was significantly higher in the SPL5 group than those in the other treatment groups (*p* < 0.05; Fig. 2C).

### The cecal microbial population at phylum and genus levels

**Fig. 3:**
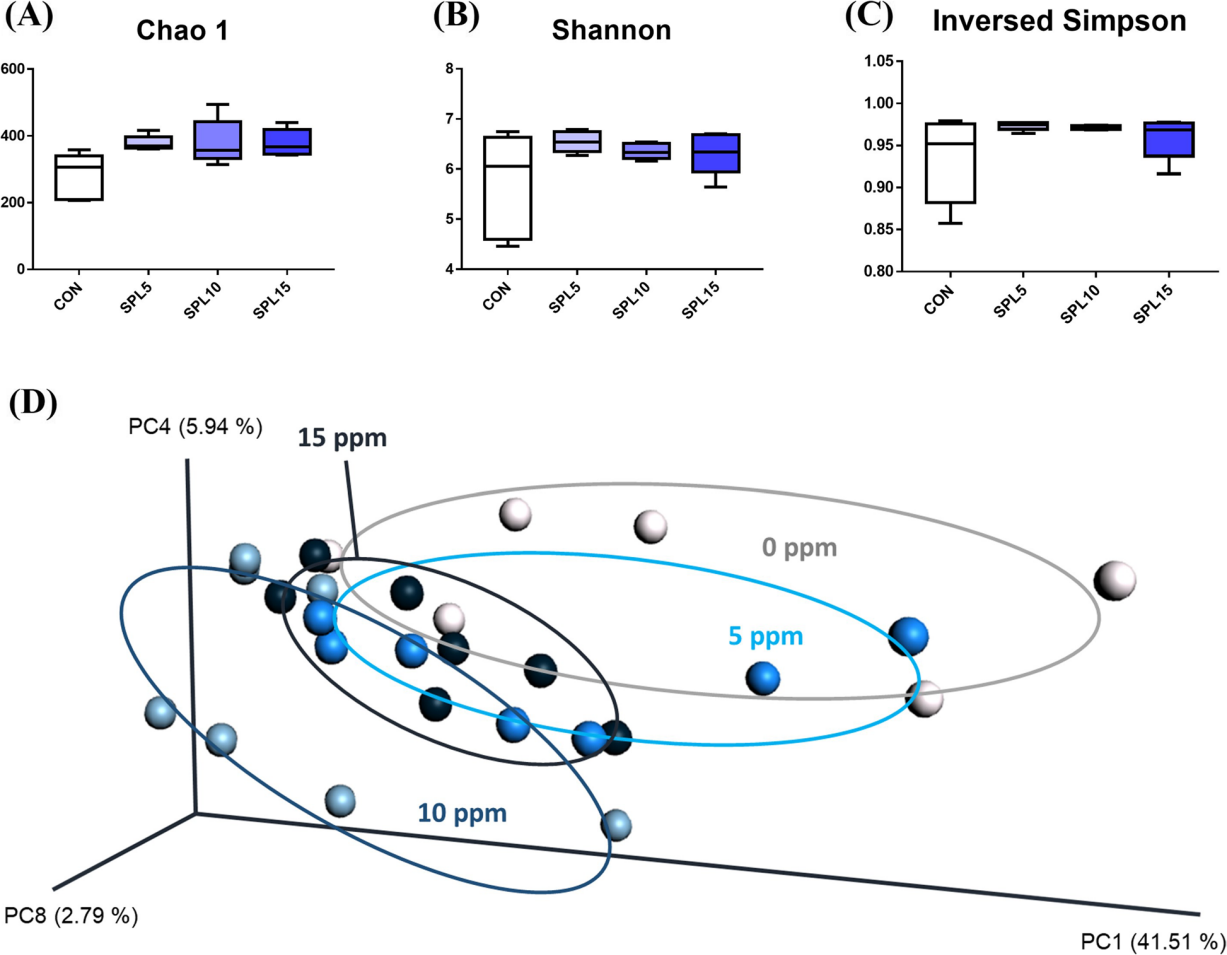
Cecal microbial community of broiler chickens fed experimental diets. **A**-**C** Dietary effects of bambermycin and sophorolipid on species diverstiy indexes (Chao1, Shannon, and inversed simpson); **D** Principal component Analysis ordination plots of microbial communities in the CON, SPL5, SPL10, and SPL15 groups based on the Jensen-Shannon distance metric. Treatment groups: CON, control group fed with basal diet; SPL5, group fed with 5 mg/kg of SPL supplemented diet; SPL10, group fed with 10 mg/kg of sophorolipid-supplemented diet; SPL15, group fed with 15 mg/kg of sophorolipid-supplemented diet

The Chao1 index of the gut microbiome was significantly increased in all dosages of SPL supplemented groups than in the CON group (*p* < 0.05; Fig. 3A); however, the Shannon and inverted Simpson indices were similar (Fig. 3B and C). Additionally, the microbial community of SPL5 and SPL15 groups were slightly separated from that of CON group, and SPL10 group showed completely separated microbial population from CON group (Fig. 3D).

**Fig. 4:**
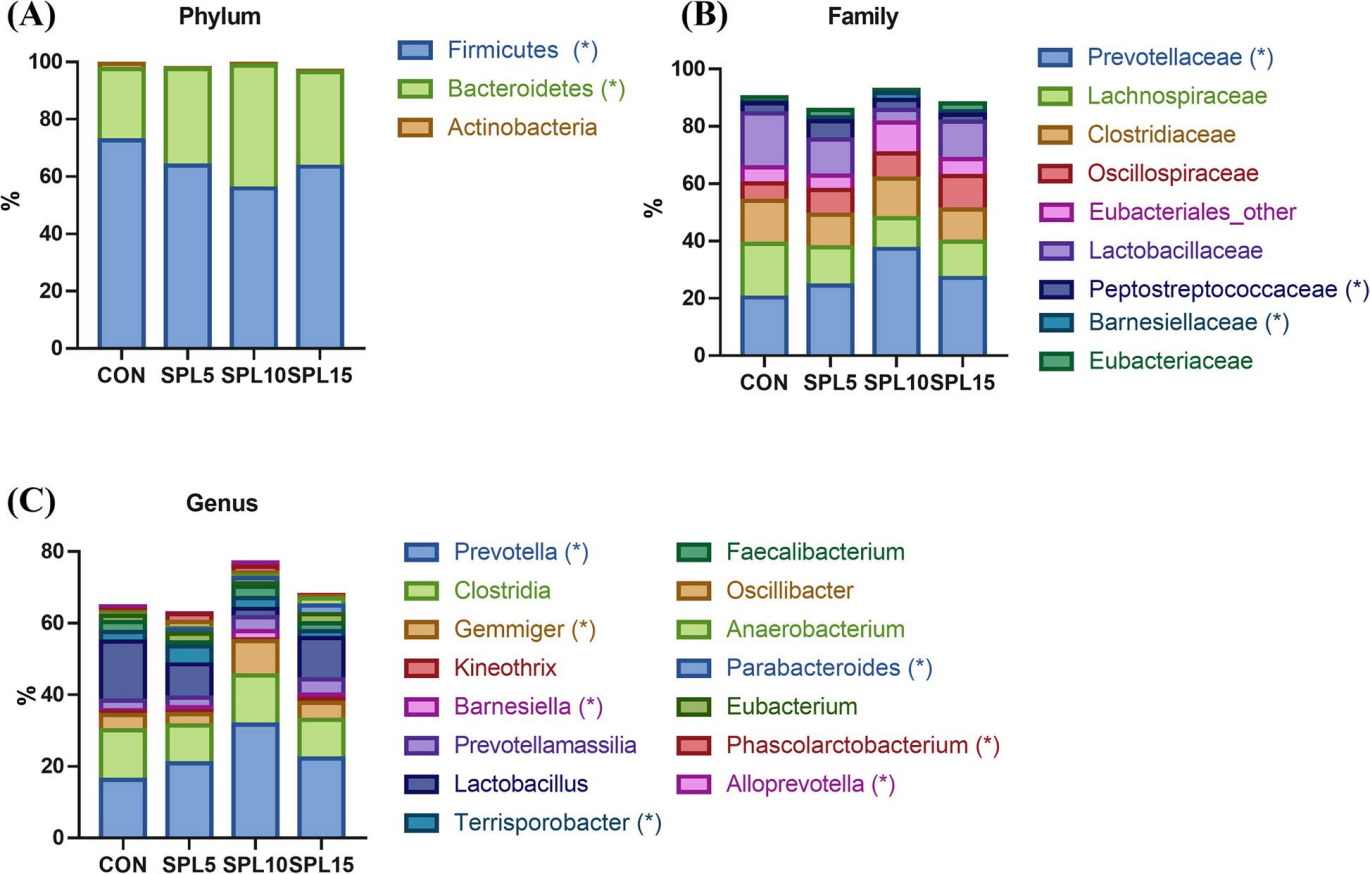
Gut microbiota population of pigs fed with experimental diets. **A** Intestinal microflora at phylum level; **B** Intestinal microflora at family level; **C** Intestinal microflora at genus level. *P < 0.05 compared with CON group. Treatment groups: CON, control group fed with basal diet; SPL5, group fed with 5 mg/kg of SPL supplemented diet; SPL10, group fed with 10 mg/kg of sophorolipid-supplemented diet; SPL15, group fed with 15 mg/kg of sophorolipid-supplemented diet

The proportions of *Bacteroidetes* and *Firmicutes* at the phylum level were significantly higher and lower, respectively, in the SPL10 group relative to the other treatments (*p* < 0.05; Fig. 4A). Furthermore, at the family level, the *Prevotellaceae*, *Peptostreptococcaceae*, and *Parnesiellaceae* populations were significantly highest in the SPL10 group among treatments (*p* < 0.05; Fig. 4B). Moreover, the proportions of *Prevotella*, *Gemmiger*, *Barnesiella, Parabacteroides*, *Phascolarctobacterium*, and *Alloprevotella* were significantly upregulated in the SPL10 group compared to the other treatment groups (*p* < 0.05; Fig. 4C).

### Cecal short-chain fatty acids (SCFA) concentration

**Fig. 5:**
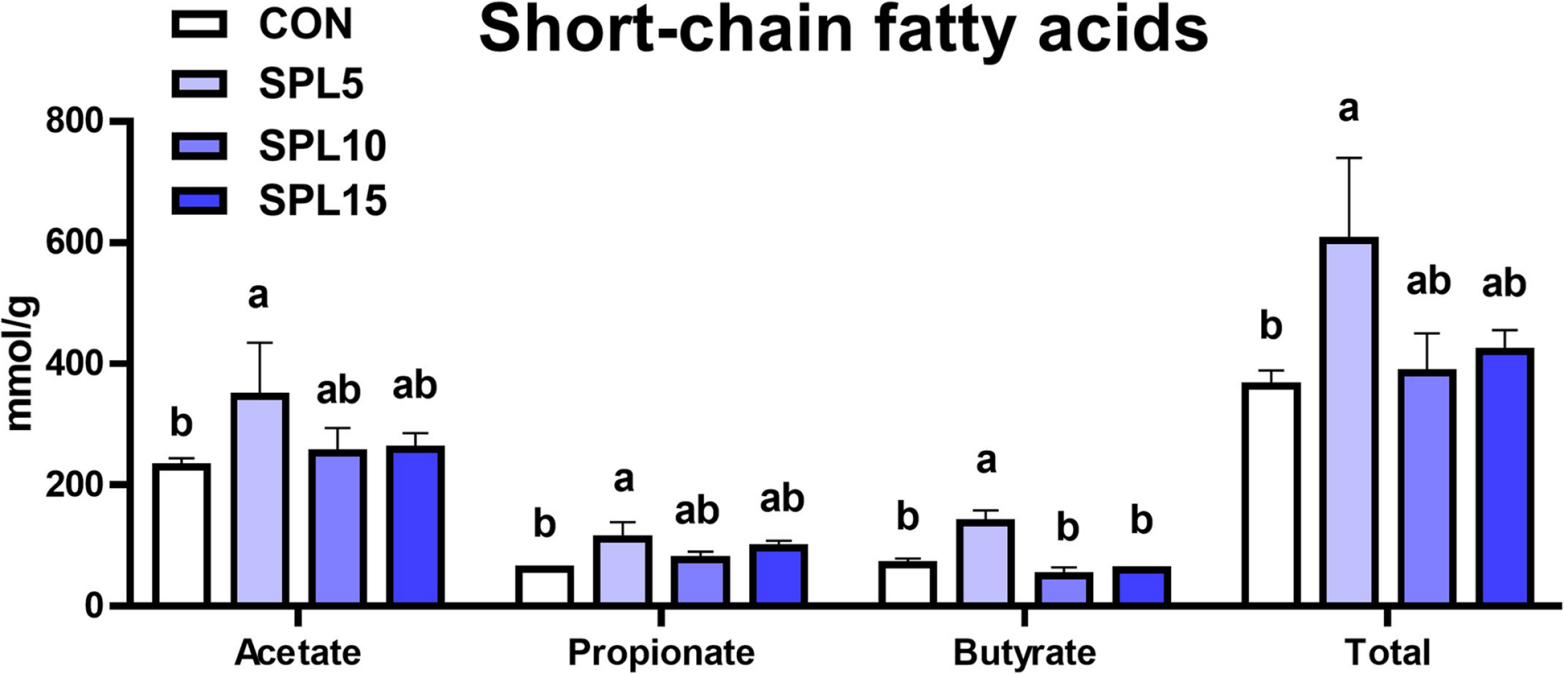
SCFA concentration (acetate, propionate, butyrate, and total) in cecum of pigs fed experimental diets. ^a,b^ Mean values within a row have different superscript letters were significantly different (*P* < 0.05). Treatment groups: CON, control group fed with basal diet; SPL5, group fed with 5 mg/kg of SPL supplemented diet; SPL10, group fed with 10 mg/kg of sophorolipid-supplemented diet; SPL15, group fed with 15 mg/kg of sophorolipid-supplemented diet

All SCFA types (acetate, propionate, and butyrate) were significantly increased in the SPL5 group compared with those in the CON group (*p* < 0.05; Fig. 5). However, 10 and 15 ppm of SPL supplementation did not change the SCFA concentration.

## Discussion

Previous studies have demonstrated that dietary SPL could enhance intestinal wound healing capacity by upregulating mucus production in cell, rat, and poultry models [24, 25]. MUC2 is the core protein of the membrane-linked mucus layer, and recent studies have demonstrated that MUC2 plays an important role in the gut defense system by lowering inflammation and improving gut integrity [26]. In addition, dietary SPL supplementation could increase the expression levels of jejunal and cecal MUC2 and the number of goblet cells in piglets. An improved gut mucus layer could protect against weaning stresses, including growth retardation and diarrhea.

*Prevotella* is the most dominant genus in the gastrointestinal tract of pigs, and its population gradually increases after weaning [27]. Moreover, Wright et al. demonstrated that *Prevotella* could utilize the mucus layer due to its mucin-desulfating glycosidase production [28]. Thus, the present study results suggest that dietary SPL supplementation could enhance the mucus layer, which would, in turn, dramatically increase the abundance of *Prevotella* after weaning. Moreover, the gut microbial complexity of animals considerably increases as they grow after weaning [29]. In the present study, dietary SPL supplementation increased the microbial richness and Chao1 indices; notably, the SPL10 treatment group had a completely separate microbial community relative to the CON group. All together our study demonstrated that optimal SPL supplementation in pig feed could improve the gastrointestinal defense system by reinforcing mucus thickness, resulting in an increase in the *Prevotella* population in piglet gut microbiota.

In the present study, piglets consumed similar amounts of feed, and the feed efficiency was also similar during the entire experimental period. However, the overall ADG of pigs fed with the SPL-supplemented diet increased quadratically in a dose-dependent manner. Our previous study with early-weaned rats demonstrated that dietary SPL supplementation (10 mg/kg) could accelerate rat growth after early weaning by improving the gut remodeling potential [24]. On the other hand, Li et al. suggested that weaning stress could agitate the intestinal microbial population, resulting in a significant reduction in *Bacteroidetes*, as well as sharp declines in *Prevotellaceae,* particularly *Prevotella* populations [30]. Similarly, a comparable microbial shift at weaning was also found in the present study, with the SPL10 treatment significantly increasing the proportion of the *Prevotella* population. Additionally, March et al. reported that *Prevotella-*enriched pigs exhibited increased growth performance, which might be due to the ability of *Prevotella* to ferment complex polysaccharides in feed [31]. In 2017, Chen et al. demonstrated intestinal *Prevotella* could contribute to high production of propionate with fructooligosaccharide, sorghum, and corn [32]. Moreover, these results could propose that the high corn portion in feed could aid the functional activity of *Prevotella* in gut of weaning piglets, however further studies will be needed. Here, we propose that dietary supplementation with 10 mg/kg SPL could improve gut bacterial modulation, which could increase ADG. Nevertheless, 5 and 15 mg/kg SPL showed an insufficient modulation in gut microbiome communities, which could be indicated that the 10 mg/kg of SPL would be an appropriate dosage in swine feed.

Albumin is a protein made by the liver that helps maintain fluid in the blood of animals by acting as a transport protein that binds to various ligands [33]. Therefore, a high serum albumin concentration could reflect the dehydration status of an animal and might indicate severe diarrhea [34]. In the present study, we found a significantly lower concentration of albumin in the sera of piglets fed with the SPL-supplemented diet than that of pigs in the CON group—the albumin concentrations of pigs in the SPL groups were within the normal range. This result indicated that dietary SPL could prevent dehydration due to weaning diarrhea by strengthening the gut defense system, a possible consequence of the dramatic increase in *Prevotella* abundance. Consistent with our study, various studies demonstrated that a higher abundance of intestinal *Prevotella* populations might have a protective effect against weaning diarrhea [35, 36]. Thus, dietary SPL supplementation could improve gut resilience by ameliorating the immune response, strengthening tight junctions, and fortifying mucus production.

Decreased expression levels of pro-inflammatory cytokines without regulation of anti-inflammatory cytokines demonstrated that dietary SPL supplementation in the post-weaning period could downregulate the local immune response against external stresses. This phenomenon suggests that the dramatic increase in the *Gemmiger* population might potentially protect against inflammation after weaning. Forbes et al. demonstrated that the population of *Gemmiger* was significantly lower in patients with inflammatory bowel disease and consistently enriched in healthy people [37]. Moreover, *Gemmiger* spp. are among the candidate microbes in probiotic formulations for colorectal cancer [38]. Therefore, these results indicate that the addition of SPL to pig feed could ameliorate the immune response in the gut by increasing *Gemmiger* abundance in the intestinal microbial community.

Zhang et al. demonstrated that an increase in propionate in the hindgut could contribute to intestinal development and enhancement of jejunal barrier integrity [39]. Furthermore, the increase in butyrate concentration in the large intestine could not only enhance animal growth but also improve the intestinal mucosal environment in weaned piglets and growing pigs [40, 41]. This might be due to the use of butyric acid as the main energy fuel for enterocytes in the large intestine [42]. In our study, optimal dietary SPL addition could increase the concentration of all SCFAs by increasing the *Prevotella* population in the cecum. Various studies have demonstrated that *Prevotella* showed high positive correlations with acetate, propionate, and butyrate production [43–45]. Moreover, Diao et al. proposed that increased gastric infusion of SCFA could decrease apoptosis in enterocytes by improving gut barrier function in weaned piglets [46]. Collectively, these results suggest that dietary SPL supplementation could aid the infusion of SCFAs into enterocytes and upregulate the restoration of gut integrity after weaning.

## Conclusions

Sophorolipid supplementation in pig feed could control the gut microbiota population by improving the mucus layer. In addition, the upregulated infusion of SCFAs into enterocytes could strengthen tight junctions, increase mucin secretion capacity, and ameliorate immune responses. Our study indicated that 10 mg/kg SPL could be used as a feed additive in the post-weaning period of piglets for quick restoration of intestinal barrier function and integrity.

## Data Availability

The whole datasets in current study are available from first authors and corresponding author on reasonable requests.

## Abbreviations

ADG: Average daily gain
ADFI: Average daily feed intake
BW: Body weight
CLDN1: Claudin 1
FE: Feed efficiency
GAPDH: Glyceraldegyde-3-phosphate dehydrogenase
GC-MS: Gas chromatography-mass spectrometry
IFN-γ: Interferon-γ
IL-8: Interleukin-8
IL-10: Interleukin-10
MUC2: Mucin 2
OCLD: Occludin
SCFA: Short-chain fatty acid
SPL: Sophorolipid
TNF-α: Tumor necrosis factor-α
ZO-1: Zonula occludens-1
